# Supplementary material for: Malaria infection and its association with socio-demographics, long lasting insecticide nets usage and hematological parameters among adolescent patients in rural Southwestern Nigeria
Source: PLoS One. 2023 Jul 14;18(7):e0287723. doi: 10.1371/journal.pone.0287723 (PMC10348556; doi:10.1371/journal.pone.0287723)
Supplement: S1 File — (DOCX) [file pone.0287723.s001.docx]

**DATA ON MALARIA PARASITAEMIA AND ITS ASSOCIATION WITH SOCIODEMOGRAPHICS, LL ITNs USAGE, AND HAEMATOLOGICAL PARAMETERS AMONG ADOLESCENTS PATIENTS IN RURAL SOUTHWESTERN NIGERIA.**

**KEY: SEX (M/F), AGE, MP (Malaria Parasite), DMP (Density of malaria parasites) OCC (Occupation),EDU (Education), DOM(Domicile), PCV, Rhesus factor (RD), Genotype (GEN), Blood group (BG), Ownership of ITNs (OWS), Sleeping under ITNs (SLITNS). P(positive), N(Negative), ST(Student), AT (Artisan), TD(Trader), FM (Farmer), NO (None), PM (Primary), SD (Secondary), TER(Tertiary), RU(Rural), UR (Urban),Neg(Negative), Pos (Positive) .**

**DMP (Mild (<1000), Moderate (1000-9999), Severe (>10000)**

| S/N | SEX | MP | AGE | DMP | OCC | ED | DOM | PCV | RD | GET | BG | OWN | SLI |  |  |  |
| --- | --- | --- | --- | --- | --- | --- | --- | --- | --- | --- | --- | --- | --- | --- | --- | --- |
| 1 | M | P | 10 | 11000 | ST | Pri | Rur | 24 | Neg | AA | B | NO | NO |  |  |  |
| 2 | F | P | 12 | 600 | ST | Pri | Rur | 23 | Pos | AA | O | Yes | Yes |  |  |  |
| 3 | M | P | 18 | 450 | Fam | Pri | Rur | 29 | Pos | AA | A | NO | NO |  |  |  |
| 4 | F | P | 14 | 4000 | ST | Sec | Urb | 32 | Pos | AA | B | Yes | Yes |  |  |  |
| 5 | M | N | 12 | NIL | ST | Pri | Rur | 22 | Pos | AC | O | yes | Yes |  |  |  |
| 6 | F | N | 18 | NIL | TD | Ter | Urb | 39 | Pos | AA | O | Yes | Yes |  |  |  |
| 7 | M | P | 14 | 600 | ST | Sec | Urb | 40 | Pos | AS | A | Yes | NO |  |  |  |
| 8 | F | P | 16 | 530 | ST | Sec | Rur | 36 | Pos | AA | A | NO | NO |  |  |  |
| 9 | M | P | 12 | 2500 | ST | Pri | Rur | 24 | Pos | AA | AB | NO | NO |  |  |  |
| 10 | F | P | 14 | 800 | ST | Pri | Rur | 37 | Pos | AS | O | Yes | NO |  |  |  |
| 11 | M | N | 16 | NIL | ST | Ter | Urb | 40 | Neg | AA | B | Yes | Yes |  |  |  |
| 12 | F | N | 12 | NIL | AT | Pri | Rur | 40 | Pos | AA | O | Yes | Yes |  |  |  |
| 13 | M | P | 16 | 12000 | TD | Pri | Rur | 39 | Pos | AA | O | NO | NO |  |  |  |
| 14 | M | P | 10 | 890 | ST | Pri | Rur | 19 | Pos | AA | O | Yes | NO |  |  |  |
| 15 | F | P | 19 | 25 | ST | Ter | Urb | 37 | Pos | AA | O | Yes | Yes |  |  |  |
| 16 | F | P | 13 | 1400 | ST | Pri | Rur | 28 | Pos | AA | B | NO | NO |  |  |  |
| 17 | M | P | 18 | 700 | ST | Sec | Rur | 39 | Pos | AA | B | Yes | Yes |  |  |  |
| 18 | M | P | 14 | 10500 | ST | Sec | Rur | 28 | Pos | AS | O | Yes | NO |  |  |  |
| 19 | F | N | 11 | NIL | ST | Pri | Rur | 29 | Pos | AA | O | Yes | Yes |  |  |  |
| 20 | M | P | 19 | 4800 | Fam | No | Rur | 28 | Pos | AA | A | NO | NO |  |  |  |
| 21 | F | P | 17 | 760 | ST | Sec | Rur | 39 | Neg | AA | A | Yes | NO |  |  |  |
| 22 | M | N | 14 | NIL | AT | Pri | Urb | 19 | Pos | SS | AB | Yes | Yes |  |  |  |
| 23 | F | P | 13 | 14000 | ST | Pri | Rur | 29 | Pos | AA | O | Yes | Yes |  |  |  |
| 24 | M | P | 15 | 840 | ST | Sec | Rur | 38 | Neg | AA | O | Yes | NO |  |  |  |
| 25 | F | N | 12 | NIL | TD | Pri | Urb | 24 | Pos | SC | O | Yes | Yes |  |  |  |
| 26 | M | P | 18 | 12800 | Fam | none | Rur | 42 | Pos | AA | O | NO | NO |  |  |  |
| 27 | F | N | 14 | NIL | ST | Ter | Urb | 44 | Pos | AA | B | Yes | Yes |  |  |  |
| 28 | F | P | 12 | 2800 | ST | Pri | Rur | 22 | Pos | AA | O | NO | NO |  |  |  |
| 29 | F | P | 18 | 670 | ST | Ter | Urb | 38 | Pos | AS | B | Yes | Yes |  |  |  |
| 30 | M | P | 14 | 960 | ST | Sec | Rur | 29 | Pos | AA | O | Yes | Yes |  |  |  |
| 31 | F | N | 12 | NIL | TD | Pri | Urb | 36 | Pos | AA | AB | Yes | Yes |  |  |  |
| 32 | M | P | 17 | 2300 | ST | Sec | Rur | 39 | Pos | AS | A | Yes | NO |  |  |  |
| 33 | F | P | 18 | 560 | ST | Ter | Urb | 38 | Pos | AA | A | Yes | Yes |  |  |  |
| 34 | M | P | 12 | 6500 | ST | Pri | Rur | 28 | Pos | AA | B | Yes | NO |  |  |  |
| 35 | F | P | 14 | 975 | TD | Pri | Rur | 32 | Pos | AA | O | NO | NO |  |  |  |
| 36 | M | P | 19 | 874 | Fam | Pri | Rur | 32 | Pos | AA | O | NO | NO |  |  |  |
| 37 | F | P | 15 | 920 | AT | PrI | Urb | 36 | Pos | AA | B | Yes | Yes |  |  |  |
| 38 | M | P | 14 | 14000 | ST | Sec | Rur | 38 | Pos | AA | O | Yes | Yes |  |  |  |
| 39 | F | P | 12 | 540 | ST | Pri | Rur | 26 | Pos | AA | A | NO | NO |  |  |  |
| 40 | M | N | 19 | NIL | ST | Ter | Urb | 40 | Pos | AA | AB | Yes | Yes |  |  |  |
| 41 | F | P | 14 | 7800 | ST | Sec | Rur | 34 | Pos | AA | O | Yes | NO |  |  |  |
| 42 | M | P | 13 | 580 | ST | Pri | Rur | 27 | Neg | AS | A | NO | NO |  |  |  |
| 43 | F | N | 12 | NIL | AT | Pri | Rur | 37 | Pos | AA | O | Yes | Yes |  |  |  |
| 44 | M | P | 16 | 4600 | ST | Sec | Rur | 38 | Pos | AA | B | Yes | Yes |  |  |  |
| 45 | F | P | 17 | 740 | ST | Sec | Rur | 36 | Pos | AA | O | NO | NO |  |  |  |
| 46 | M | N | 13 | NIL | TD | Pri | Urb | 32 | Pos | AA | B | Yes | Yes |  |  |  |
| 47 | F | P | 16 | 13600 | TD | NO | Rur | 38 | Pos | AA | O | NO | NO |  |  |  |
| 48 | M | P | 14 | 590 | ST | Pri | Rur | 39 | Pos | AA | AB | NO | NO |  |  |  |
| 49 | F | N | 15 | NIL | ST | Pri | Rur | 38 | Neg | AS | O | yes | Yes |  |  |  |
| 50 | M | P | 18 | 2560 | AT | NO | Rur | 41 | Pos | AA | O | Yes | NO |  |  |  |
| 51 | F | P | 11 | 950 | ST | Pri | Rur | 23 | Pos | AA | A | NO | NO |  |  |  |
| 52 | M | N | 16 | NIL | ST | Sec | Urb | 43 | Pos | AS | A | Yes | Yes |  |  |  |
| 53 | M | P | 11 | 1800 | ST | Pri | Rur | 22 | Pos | AA | A | NO | NO |  |  |  |
| 54 | F | P | 14 | 760 | ST | Pri | Rur | 32 | Pos | AA | B | NO | NO |  |  |  |
| 55 | M | P | 10 | 650 | ST | Pri | Rur | 18 | Pos | SS | O | Yes | NO |  |  |  |
| 56 | F | N | 18 | NIL | ST | Ter | Urb | 30 | Pos | AC | A | Yes | Yes |  |  |  |
| 57 | M | P | 12 | 8600 | ST | Pri | Rur | 28 | Pos | AA | B | NO | NO |  |  |  |
| 58 | F | P | 13 | 745 | AT | NO | Rur | 38 | Pos | AA | O | Yes | NO |  |  |  |
| 59 | M | P | 15 | 12500 | ST | Sec | Rur | 39 | Pos | AA | O | Yes | NO |  |  |  |
| 60 | F | P | 16 | 658 | ST | Sec | Rur | 38 | Neg | AA | O | Yes | Yes |  |  |  |
| 61 | M | P | 17 | 963 | ST | Sec | Rur | 42 | Pos | AA | A | NO | NO |  |  |  |
| 62 | F | N | 10 | NIL | ST | Pri | Rur | 34 | Pos | AA | B | Yes | Yes |  |  |  |
| 63 | M | N | 14 | NIL | ST | Sec | Urb | 36 | Pos | AA | A | Yes | Yes |  |  |  |
| 64 | F | P | 16 | 13100 | TD | Pri | Rur | 38 | Pos | AA | A | NO | NO |  |  |  |
| 65 | M | P | 19 | 560 | TD | Ter | Rur | 40 | Neg | AS | O | Yes | NO |  |  |  |
| 66 | F | P | 12 | 740 | ST | Pri | Rur | 36 | Pos | AA | B | Yes | NO |  |  |  |
| 67 | M | P | 13 | 9800 | ST | Pri | Rur | 27 | Pos | AA | B | Yes | Yes |  |  |  |
| 68 | F | N | 19 | NIL | ST | Ter | Urb | 40 | Pos | AS | O | Yes | Yes |  |  |  |
| 69 | M | N | 18 | NIL | TD | NO | Rur | 41 | Neg | AA | AB | Yes | Yes |  |  |  |
| 70 | F | P | 10 | 4600 | ST | Pri | Rur | 19 | Pos | AA | O | Yes | NO |  |  |  |
| 71 | M | P | 16 | 850 | ST | Sec | Rur | 40 | Pos | AA | O | NO | NO |  |  |  |
| 72 | M | P | 12 | 990 | ST | Pri | Rur | 23 | Pos | AS | A | Yes | Yes |  |  |  |
| 73 | F | P | 11 | 7500 | ST | Pri | Rur | 28 | Pos | AA | A | Yes | NO |  |  |  |
| 74 | M | N | 14 | NIL | ST | Sec | Urb | 34 | Pos | AA | B | Yes | Yes |  |  |  |
| 75 | F | N | 15 | NIL | TD | Pri | Rur | 39 | Pos | AA | O | Yes | Yes |  |  |  |
| 76 | M | P | 17 | 963 | ST | Ter | Rur | 40 | Pos | AA | B | NO | NO |  |  |  |
| 77 | F | P | 10 | 4200 | ST | Pri | Rur | 24 | Pos | AA | O | Yes | NO |  |  |  |
| 78 | M | P | 14 | 8000 | AT | Pri | Rur | 28 | Pos | AA | O | NO | NO |  |  |  |
| 79 | F | P | 17 | 860 | TD | No | Rur | 40 | Pos | AS | O | Yes | NO |  |  |  |
| 80 | M | N | 10 | NIL | ST | Pri | Rur | 26 | Pos | AA | A | Yes | Yes |  |  |  |
| 81 | F | N | 16 | NIL | ST | Ter | Urb | 38 | Pos | AA | AB | Yes | Yes |  |  |  |
| 82 | M | P | 13 | 740 | ST | Pri | Rur | 27 | Pos | AS | O | Yes | Yes |  |  |  |
| 83 | F | P | 12 | 10300 | ST | Pri | Rur | 32 | Pos | AA | B | NO | NO |  |  |  |
| 84 | M | P | 15 | 450 | ST | Sec | Rur | 36 | Pos | AA | A | Yes | NO |  |  |  |
| 85 | F | P | 16 | 460 | ST | Sec | Rur | 29 | Pos | AA | O | NO | NO |  |  |  |
| 86 | M | N | 12 | 340 | AT | Pri | Urb | 34 | Pos | AS | AB | Yes | Yes |  |  |  |
| 87 | F | N | 14 | NIL | TD | Pri | Rur | 29 | Pos | AA | O | Yes | Yes |  |  |  |
| 88 | M | P | 16 | 854 | ST | Sec | Urb | 38 | Neg | AS | B | Yes | Yes |  |  |  |
| 89 | M | P | 13 | 5800 | ST | Pri | Rur | 29 | Pos | AA | O | Yes | NO |  |  |  |
| 90 | F | P | 12 | 3800 | ST | Pri | Rur | 23 | Neg | AA | A | NO | NO |  |  |  |
| 91 | M | N | 15 | 963 | TD | Pri | Rur | 38 | Pos | AA | A | Yes | Yes |  |  |  |
| 92 | F | P | 12 | 14000 | ST | Pri | Rur | 26 | Pos | AS | O | Yes | Yes |  |  |  |
| 93 | M | P | 17 | 670 | Fam | Pri | Rur | 30 | Pos | AA | B | NO | NO |  |  |  |
| 94 | F | N | 12 | NIL | ST | Pri | Urb | 38 | Pos | AA | B | Yes | Yes |  |  |  |
| 95 | M | P | 14 | 4300 | AT | Pri | Rur | 28 | Pos | AS | O | NO | NO |  |  |  |
| 96 | F | P | 13 | 647 | ST | Sec | Rur | 27 | Pos | AA | AB | NO | NO |  |  |  |
| 97 | M | N | 19 | NIL | ST | Ter | Urb | 24 | Neg | SS | O | Yes | Yes |  |  |  |
| 98 | M | P | 12 | 8600 | ST | Pri | Rur | 28 | Pos | AA | O | Yes | NO |  |  |  |
| 99 | F | P | 15 | 760 | TD | Pri | Rur | 38 | Pos | AA | B | NO | NO |  |  |  |
| 100 | M | P | 19 | 748 | ST | Ter | Rur | 19 | Pos | AC | A | Yes | NO |  |  |  |
| 101 | F | N | 12 | NIL | ST | Sec | Urb | 34 | Neg | AA | O | Yes | Yes |  |  |  |
| 102 | M | N | 11 | NIL | ST | Pri | Urb | 38 | Pos | AA | B | Yes | Yes |  |  |  |
| 103 | F | P | 17 | 3900 | ST | Ter | Rur | 39 | Pos | AS | A | NO | NO |  |  |  |
| 104 | M | P | 15 | 650 | ST | Sec | Rur | 38 | Pos | AS | O | NO | NO |  |  |  |
| 105 | F | P | 14 | 11800 | ST | Sec | Rur | 36 | Pos | AA | O | NO | NO |  |  |  |
| 106 | M | P | 16 | 639 | ST | Sec | Urb | 18 | Neg | SC | O | NO | NO |  |  |  |
| 107 | M | N | 12 | NIL | ST | Pri | Rur | 38 | Pos | AA | A | Yes | Yes |  |  |  |
| 108 | F | P | 18 | 7300 | TD | Pri | Rur | 40 | Pos | AA | AB | NO | NO |  |  |  |
| 109 | M | P | 10 | 6500 | ST | Pri | Rur | 23 | Pos | AA | B | Yes | NO |  |  |  |
| 110 | F | P | 12 | 860 | ST | Pri | Rur | 26 | Pos | AA | O | NO | NO |  |  |  |
| 111 | M | P | 15 | 690 | AT | Pri | Rur | 38 | Pos | AS | A | Yes | NO |  |  |  |
| 112 | F | P | 11 | 8400 | ST | Pri | Rur | 24 | Pos | AA | B | NO | NO |  |  |  |
| 113 | M | N | 18 | NIL | ST | Ter | Urb | 40 | Pos | AA | AB | Yes | Yes |  |  |  |
| 114 | F | N | 13 | NIL | ST | Sec | Urb | 33 | Pos | AA | O | Yes | Yes |  |  |  |
| 115 | M | P | 11 | 10200 | ST | Pri | Rur | 27 | Pos | AA | O | NO | NO |  |  |  |
| 116 | F | P | 15 | 680 | ST | Sec | Rur | 34 | Pos | AS | A | NO | NO |  |  |  |
| 117 | M | P | 18 | 760 | ST | Sec | Rur | 40 | Pos | AA | O | Yes | Yes |  |  |  |
| 118 | F | P | 12 | 4600 | ST | Pri | Rur | 28 | Pos | AA | O | Yes | Yes |  |  |  |
| 119 | M | N | 14 | NIL | TD | NO | Rur | 37 | Pos | AA | A | NO | NO |  |  |  |
| 120 | F | N | 19 | NIL | TD | Ter | Urb | 18 | Neg | SS | AB | Yes | Yes |  |  |  |
| 121 | M | P | 16 | 560 | ST | Sec | Urb | 32 | Pos | AS | A | Yes | Yes |  |  |  |
| 122 | F | P | 10 | 9400 | ST | Pri | Rur | 24 | Pos | AA | O | NO | NO |  |  |  |
| 123 | M | P | 17 | 600 | AT | Pri | Rur | 39 | Pos | AA | B | NO | NO |  |  |  |
| 124 | F | P | 15 | 2100 | ST | Sec | Rur | 38 | Pos | AS | O | NO | NO |  |  |  |
| 125 | M | P | 13 | 760 | ST | Pri | Rur | 29 | Pos | AA | O | Yes | NO |  |  |  |
| 126 | F | N | 16 | NIL | TD | Pri | Rur | 36 | Pos | AA | A | Yes | Yes |  |  |  |
| 127 | M | N | 16 | NIL | ST | Ter | Urb | 36 | Neg | AA | O | Yes | Yes |  |  |  |
| 128 | M | P | 12 | 3900 | ST | Pri | Rur | 32 | Pos | AA | O | Yes | NO |  |  |  |
| 129 | F | P | 10 | 10900 | ST | Pri | Rur | 19 | Pos | AA | B | NO | NO |  |  |  |
| 130 | M | P | 15 | 680 | AT | Pri | Rur | 34 | Pos | AA | A | NO | NO |  |  |  |
| 131 | F | P | 14 | 780 | ST | Sec | Rur | 33 | Pos | AA | A | NO | NO |  |  |  |
| 132 | M | N | 18 | NIL | TD | Ter | Urb | 24 | Pos | AC | B | Yes | Yes |  |  |  |
| 133 | F | N | 11 | NIL | ST | Pri | Urb | 30 | Pos | AA | O | Yes | Yes |  |  |  |
| 134 | M | P | 13 | 5700 | ST | Pri | Rur | 30 | Neg | AA | B | NO | NO |  |  |  |
| 135 | F | P | 16 | 500 | ST | Sec | Rur | 30 | Pos | AA | O | NO | NO |  |  |  |
| 136 | M | P | 10 | 3600 | ST | Pri | Rur | 30 | Pos | AS | O | Yes | NO |  |  |  |
| 137 | F | P | 19 | 450 | Fam | No | Rur | 32 | Pos | AA | O | NO | NO |  |  |  |
| 138 | M | N | 12 | NIL | AT | Pri | Rur | 28 | Pos | AA | AB | NO | NO |  |  |  |
| 139 | M | P | 14 | 860 | ST | Sec | Urb | 34 | Pos | AA | O | Yes | Yes |  |  |  |
| 140 | F | P | 17 | 780 | AT | Pri | Rur | 34 | Pos | AS | O | Yes | NO |  |  |  |
| 141 | F | P | 18 | 1300 | TD | Pri | Rur | 30 | Pos | AA | A | NO | NO |  |  |  |
| 142 | M | P | 10 | 560 | ST | Pri | Rur | 26 | Pos | AA | A | NO | NO |  |  |  |
| 143 | F | P | 14 | 3700 | ST | Sec | Rur | 32 | Pos | AA | B | Yes | NO |  |  |  |
| 144 | M | N | 16 | NIL | TD | Pri | Urb | 22 | Pos | SC | B | Yes | Yes |  |  |  |
| 145 | F | N | 12 | NIL | ST | Pri | Rur | 28 | Pos | AS | O | Yes | Yes |  |  |  |
| 146 | M | P | 15 | 450 | Fam | Pri | Rur | 30 | Pos | AA | O | NO | NO |  |  |  |
| 147 | F | P | 11 | 900 | TD | Pri | Rur | 18 | Pos | AA | O | NO | NO |  |  |  |
| 148 | M | P | 10 | 8500 | ST | Pri | Rur | 22 | Pos | AS | A | NO | NO |  |  |  |
| 149 | F | P | 18 | 600 | ST | Ter | Urb | 34 | Pos | AA | AB | NO | NO |  |  |  |
| 150 | M | N | 13 | NIL | AT | Pri | Rur | 36 | Pos | AA | O | Yes | Yes |  |  |  |
| 151 | M | N | 19 | NIL | AT | Ter | Urb | 42 | Pos | AS | A | Yes | Yes |  |  |  |
| 152 | F | P | 12 | 16000 | ST | Pri | Rur | 30 | Pos | AA | A | Yes | NO |  |  |  |
| 153 | M | P | 14 | 650 | ST | Sec | Rur | 32 | Neg | AA | A | NO | NO |  |  |  |
| 154 | F | N | 16 | NIL | TD | Pri | Urb | 22 | Pos | SS | A | Yes | Yes |  |  |  |
| 155 | M | P | 12 | 700 | ST | Pri | Rur | 32 | Pos | AA | O | Yes | Yes |  |  |  |
| 156 | F | P | 17 | 860 | ST | Sec | Rur | 27 | Pos | AA | O | NO | NO |  |  |  |
| 157 | M | N | 18 | NIL | ST | Ter | Urb | 40 | Pos | AA | AB | Yes | Yes |  |  |  |
| 158 | F | P | 14 | 7400 | ST | Pri | Rur | 42 | Pos | AA | O | Yes | NO |  |  |  |
| 159 | M | P | 16 | 700 | ST | Sec | Rur | 30 | Pos | AS | A | Yes | NO |  |  |  |
| 160 | M | P | 10 | 9300 | ST | Pri | Rur | 34 | Pos | AA | B | NO | NO |  |  |  |
| 161 | F | P | 12 | 8500 | ST | Pri | Rur | 19 | Pos | AA | O | NO | NO |  |  |  |
| 162 | F | N | 15 | NIL | ST | Pri | Rur | 37 | Pos | AA | B | yes | Yes |  |  |  |
| 163 | M | N | 19 | NIL | TD | Ter | Urb | 38 | Pos | AA | O | Yes | Yes |  |  |  |
| 164 | F | P | 14 | 6700 | ST | Pri | Rur | 29 | Pos | AS | B | NO | NO |  |  |  |
| 165 | M | N | 10 | NIL | ST | Pri | Rur | 34 | Neg | AA | O | Yes | Yes |  |  |  |
| 166 | F | P | 16 | 750 | ST | Sec | Rur | 38 | Pos | AA | O | Yes | NO |  |  |  |
| 167 | M | N | 11 | NIL | ST | Pri | Rur | 28 | Pos | AA | AB | Yes | Yes |  |  |  |
| 168 | F | P | 15 | 7300 | ST | Pri | Rur | 34 | Pos | AA | O | Yes | Yes |  |  |  |
| 169 | M | P | 15 | 800 | TD | Pri | Urb | 36 | Pos | AA | AB | NO | NO |  |  |  |
| 170 | M | P | 10 | 600 | ST | Pri | Rur | 30 | Pos | AS | A | Yes | NO |  |  |  |
| 171 | F | P | 16 | 998 | AT | Sec | Rur | 40 | Pos | AA | O | NO | NO |  |  |  |
| 172 | M | P | 14 | 1600 | ST | Sec | Rur | 35 | Pos | AA | B | Yes | Yes |  |  |  |
| 173 | F | P | 18 | 760 | ST | Sec | Rur | 36 | Neg | AA | A | Yes | Yes |  |  |  |
| 174 | M | P | 10 | 13600 | ST | Pri | Rur | 30 | Pos | AA | O | NO | NO |  |  |  |
| 175 | F | N | 16 | NIL | TD | Pri | Urb | 38 | Pos | AA | A | Yes | Yes |  |  |  |
| 176 | M | P | 14 | 560 | ST | Sec | Rur | 34 | Pos | AA | O | NO | NO |  |  |  |
| 177 | M | P | 13 | 760 | ST | Sec | Urb | 34 | Pos | AA | A | Yes | Yes |  |  |  |
| 178 | F | N | 17 | NIL | ST | Sec | Urb | 40 | Pos | AA | O | Yes | Yes |  |  |  |
| 179 | M | P | 10 | 7400 | ST | Pri | Rur | 18 | Pos | AA | O | NO | NO |  |  |  |
| 180 | F | P | 19 | 740 | ST | Ter | Rur | 30 | Neg | AS | AB | Yes | Yes |  |  |  |
