# Supplementary material for: Malaria infection and its association with socio-demographics, long lasting insecticide nets usage and hematological parameters among adolescent patients in rural Southwestern Nigeria
Source: PLoS One. 2023 Jul 14;18(7):e0287723. doi: 10.1371/journal.pone.0287723 (PMC10348556; doi:10.1371/journal.pone.0287723)
Supplement: Attachment — Submitted filename: PONE-D-23-02179_review april 2023.pdf [file pone.0287723.s007.pdf]

PLOS ONE

MALARIA INFECTION AND ITS ASSOCIATION WITH SOCIO-DEMOGRAPHICS, LONG LASTING INSECTICIDE NETS USAGE AND HEMATOLOGICAL PARAMETERS AMONG ADOLESCENT PATIENTS IN RURAL SOUTHWESTERN NIGERIA

--Manuscript Draft--

**Manuscript Number:**	PONE-D-23-02179
**Article Type:**	Research Article
**Full Title:**	MALARIA INFECTION AND ITS ASSOCIATION WITH SOCIO-DEMOGRAPHICS, LONG LASTING INSECTICIDE NETS USAGE AND HEMATOLOGICAL PARAMETERS AMONG ADOLESCENT PATIENTS IN RURAL SOUTHWESTERN NIGERIA
**Short Title:**	MALARIA INFECTION AMONG ADOLESCENTS
**Corresponding Author:**	Azeez Ibrahim<br>Afe Babalola University College of Medicine and Health Sciences<br>Ido Ekiti, NIGERIA
**Keywords:**	Prevalence, associated factors, adolescents, malaria infection, rural Nigeria.
**Abstract:**	Background: There is increasing evidence suggesting that adolescents are contributing to the populations at risk of malaria. This study determined the prevalence of malaria infection among the adolescents and examined the associated determinants considering socio-demographic, Long Lasting Insecticide Nets (LLINs) usage, and hematological factors in rural Southwestern Nigeria.<br>Methods: A hospital-based cross sectional study was conducted between July 2021 and September 2022 among 180 adolescents who were recruited at a tertiary health facility in rural Southwestern Nigeria. Interviewer administered questionnaire sought information on their socio-demographics and usage of LLINs. Venous blood samples were collected and processed for malaria parasite detection, ABO blood grouping, hemoglobin genotype, and packed cell volume. Data were analyzed using SPSS version 20. A p-value <0.05 was considered statistically significant.<br>Results: The prevalence of malaria infection was 71.1% (95% CI: 68.2%-73.8%). Lack of formal education (AOR = 2.094; 95% CI: 1.288- 3.403), being a rural residence (AOR= 4.821; 95% CI: 2.805 – 8.287), not sleeping under LLINs (AOR = 1.950; 95% CI: 1.525 – 2.505), genotype AA (AOR= 3.420; 95% CI: 1.003 – 11.657), genotype AS (AOR= 3.574; 95%CI: 1.040 – 12.277), rhesus positive (AOR= 1.815; 95% CI:1.121 – 2.939), and severe anemia (AOR=1.533; 95% CI: 1.273 – 1.846) were significantly associated with malaria infection.<br>Conclusion: The study revealed a high prevalence of malaria infection among the adolescents in rural Southwestern Nigeria. There may be need to pay greater attention to adolescent populations for malaria intervention and control programs.
**Order of Authors:**	Azeez Ibrahim
	Tosin Anthony Agbesanwa
	Olayide Toyin ELEGBEDE
	Olusegun Emmanuel GABRIEL-ALAYODE
	Adewunmi Oluwaserimi AJETUNMOBI
	Ibrahim Sebutu BELLO
	Kayode Rasak ADEWOYE
	Temitope Moronkeji OLANREWAJU
	Adetunji OMONIJO
	Ebenezer Kayode ARIYIBI
	Taofeek Adedayo SANNI

Powered by Editorial Manager® and ProduXion Manager® from Aries Systems Corporation

	Shuaib Kayode AREMU
	Ayodele Kamal ALABI
**Additional Information:**	
**Question**	**Response**
**Financial Disclosure**<br><br>Enter a financial disclosure statement that describes the sources of funding for the work included in this submission. Review the submission guidelines for detailed requirements. View published research articles from *PLOS ONE* for specific examples.<br><br>This statement is required for submission and **will appear in the published article** if the submission is accepted. Please make sure it is accurate.<br><br>**Unfunded studies**<br>Enter: *The author(s) received no specific funding for this work.*<br><br>**Funded studies**<br>Enter a statement with the following details:<br>• Initials of the authors who received each award<br>• Grant numbers awarded to each author<br>• The full name of each funder<br>• URL of each funder website<br>• Did the sponsors or funders play any role in the study design, data collection and analysis, decision to publish, or preparation of the manuscript?<br>• **NO** - Include this sentence at the end of your statement: *The funders had no role in study design, data collection and analysis, decision to publish, or preparation of the manuscript.*<br>• **YES** - Specify the role(s) played.<br><br>* typeset	Self funded by authors
**Competing Interests**<br><br>Use the instructions below to enter a competing interest statement for this submission. On behalf of all authors, disclose any competing interests that could be perceived to bias this work—acknowledging all financial support and any other relevant financial or non-	No competing interest

financial competing interests.<br><br>This statement is required for submission and **will appear in the published article** if the submission is accepted. Please make sure it is accurate and that any funding sources listed in your Funding Information later in the submission form are also declared in your Financial Disclosure statement.<br><br>View published research articles from *PLOS ONE* for specific examples.<br><br>**NO authors have competing interests**<br><br>Enter: *The authors have declared that no competing interests exist.*<br><br>**Authors with competing interests**<br><br>Enter competing interest details beginning with this statement:<br><br>*I have read the journal's policy and the authors of this manuscript have the following competing interests: [insert competing interests here]*<br><br>* typeset	
**Ethics Statement**<br><br>Enter an ethics statement for this submission. This statement is required if the study involved:<br><br>• Human participants<br>• Human specimens or tissue<br>• Vertebrate animals or cephalopods<br>• Vertebrate embryos or tissues<br>• Field research<br><br>Write "N/A" if the submission does not require an ethics statement.<br><br>General guidance is provided below.<br>Consult the submission guidelines for	Approval obtained from ethical and research committee of our institution

detailed instructions. **Make sure that all information entered here is included in the Methods section of the manuscript.**<br><br>**Format for specific study types**<br><br>**Human Subject Research (involving human participants and/or tissue)**<br>• Give the name of the institutional review board or ethics committee that approved the study<br>• Include the approval number and/or a statement indicating approval of this research<br>• Indicate the form of consent obtained (written/oral) or the reason that consent was not obtained (e.g. the data were analyzed anonymously)<br><br>**Animal Research (involving vertebrate animals, embryos or tissues)**<br>• Provide the name of the Institutional Animal Care and Use Committee (IACUC) or other relevant ethics board that reviewed the study protocol, and indicate whether they approved this research or granted a formal waiver of ethical approval<br>• Include an approval number if one was obtained<br>• If the study involved *non-human primates*, add *additional details* about animal welfare and steps taken to ameliorate suffering<br>• If anesthesia, euthanasia, or any kind of animal sacrifice is part of the study, include briefly which substances and/or methods were applied<br><br>**Field Research**<br><br>Include the following details if this study involves the collection of plant, animal, or other materials from a natural setting:<br>• Field permit number<br>• Name of the institution or relevant body that granted permission	
**Data Availability**<br><br>Authors are required to make all data underlying the findings described fully available, without restriction, and from the time of publication. PLOS allows rare	Yes - all data are fully available without restriction

exceptions to address legal and ethical concerns. See the PLOS Data Policy and FAQ for detailed information.<br><br>A Data Availability Statement describing where the data can be found is required at submission. Your answers to this question constitute the Data Availability Statement and **will be published in the article**, if accepted.<br><br>**Important:** Stating 'data available on request from the author' is not sufficient. If your data are only available upon request, select 'No' for the first question and explain your exceptional situation in the text box.<br><br>Do the authors confirm that all data underlying the findings described in their manuscript are fully available without restriction?	
**Describe where the data may be found in full sentences. If you are copying our sample text, replace any instances of XXX with the appropriate details.**<br><br>• If the data are **held or will be held in a public repository**, include URLs, accession numbers or DOIs. If this information will only be available after acceptance, indicate this by ticking the box below. For example: *All XXX files are available from the XXX database (accession number(s) XXX, XXX.*).<br>• If the data are all contained **within the manuscript and/or Supporting Information files**, enter the following: *All relevant data are within the manuscript and its Supporting Information files.*<br>• If neither of these applies but you are able to provide **details of access elsewhere**, with or without limitations, please do so. For example:<br><br>*Data cannot be shared publicly because of [XXX]. Data are available from the XXX Institutional Data Access / Ethics Committee (contact via XXX) for researchers who meet the criteria for*	All relevant data are within the manuscript and its Supporting Information files.

access to confidential data.<br><br>*The data underlying the results presented in the study are available from (include the name of the third party and contact information or URL).*<br>• This text is appropriate if the data are owned by a third party and authors do not have permission to share the data.<br><br>* typeset	
Additional data availability information:	Tick here if the URLs/accession numbers/DOIs will be available only after acceptance of the manuscript for publication so that we can ensure their inclusion before publication.

Powered by Editorial Manager® and ProduXion Manager® from Aries Systems Corporation

Manuscript
Click here to access/download;Manuscript;Malaria among adolescent.docx

TITLE: MALARIA INFECTION AND ITS ASSOCIATION WITH SOCIO-DEMOGRAPHICS, LONG LASTING INSECTICIDE NETS USAGE AND HEMATOLOGICAL PARAMETERS AMONG ADOLESCENT PATIENTS IN RURAL SOUTHWESTERN NIGERIA.

Authors' Affiliation:

[1]Azeez Oyemomi IBRAHIM*, [2]Tosin Anthony Agbesanwa, [3]Adetunji OMONIJO, [1]Olayide Toyin ELEGBEDE, [1]Olusegun Emmanuel GABRIEL-ALAYODE, [1]Adewunmi Oluwaserimi AJETUNMOBI, [4]Ibrahim Sebutu BELLO, [5]Kayode Rasak ADEWOYE, [3]Temitope Moronkeji OLANREWAJU, [3]Ebenezer Kayode ARIYIBI, [5]Taofeek Adedayo SANNI, [6] Shuaib Kayode AREMU, [7]Ayodele Kamal ALABI

[1]Department of Family Medicine, Afe Babalola University, Ado-Ekiti, Ekiti State, Nigeria

[2]Department of Family Medicine, Ekiti State University, Ado Ekiti.

[3]Department of Family Medicine, Federal Teaching Hospital, Ido-Ekiti, Ekiti State

[4]Department of Family Medicine, Obafemi Awolowo University Teaching Hospital Complex, Ile-Ife, Osun State, Nigeria

[5]Department of Community Medicine, Afe Babalola University, Ado-Ekiti, Ekiti State, Nigeria

[6]Department of ENT, Afe Babalola University, Ado-Ekiti, Ekiti State, Nigeria

[7]Department of Community Medicine, Federal Teaching Hospital, Ido-Ekiti, Ekiti State, Nigeria.

*Correspondence author: [1]Azeez Oyemomi IBRAHIM. Email: ibrahimazeez1434@gmail.com

Abstract:

Background: There is increasing evidence suggesting that adolescents are contributing to the populations at risk of malaria. This study determined the prevalence of malaria infection among the adolescents and examined the associated determinants considering socio-demographic, Long Lasting Insecticide Nets (LLINs) usage, and hematological factors in rural Southwestern Nigeria.

Methods: A hospital-based cross sectional study was conducted between July 2021 and September 2022 among 180 adolescents who were recruited at a tertiary health facility in rural Southwestern Nigeria. Interviewer administered questionnaire sought information on their socio-demographics and usage of LLINs. Venous blood samples were collected and processed for malaria parasite detection, ABO blood grouping, hemoglobin genotype, and packed cell volume. Data were analyzed using SPSS version 20. A p-value <0.05 was considered statistically significant.

Results: The prevalence of malaria infection was 71.1% (95% CI: 68.2%-73.8%). Lack of formal education (AOR = 2.094; 95% CI: 1.288- 3.403), being a rural residence (AOR= 4.821; 95% CI: 2.805 – 8.287), not sleeping under LLINs (AOR = 1.950; 95% CI: 1.525 – 2.505), genotype AA (AOR= 3.420; 95% CI: 1.003 – 11.657), genotype AS (AOR= 3.574; 95%CI: 1.040 – 12.277), rhesus positive (AOR= 1.815; 95% CI:1.121 – 2.939), and severe anemia (AOR=1.533; 95% CI: 1.273 – 1.846) were significantly associated with malaria infection.

Conclusion: The study revealed a high prevalence of malaria infection among the adolescents in rural Southwestern Nigeria. There may be need to pay greater attention to adolescent populations for malaria intervention and control programs.

Key words: Prevalence, associated factors, adolescents, malaria infection, rural Nigeria.

Introduction:

Malaria remains a global disease burden, and is a major public health concern in many countries including Nigeria [1]. The disease is transmitted in humans from one person to another through the bites of infected female mosquitoes of the Anopheles [2,3]. There are five species of parasites belonging to the genus *Plasmodium*, that transmit malaria, of which *P. falciparum* being the most prevalent [2,3]. According to the 2015 WHO epidemiological record, there were about 214 million cases of malaria and 438,000 deaths [4]. However, approximately 80% of all deaths due to malaria were concentrated in just 15 countries mainly in the Africa region [2]. Despite frantic efforts and interventions targeted at its elimination, about 40% of the world population in 91 countries were still being affected by this disease [5]. In Africa, malaria is responsible for about 20-30% of hospital admissions and about 30-50% of out-patient consultations [6].

The prevalence of malaria infection among the adolescent patients varies from place to place, even within the same country, and may be due to differences in socio-demographic, environmental and climatic factors [7,8]. In Nigeria, malaria prevalence of 66.7% [9], 64.0% [10], and 58.0% [11] have been reported by previous studies. Socio-demographic factors such as age, gender, education, occupation and income, which may directly affect human exposure and treatment, and climatic factors such as temperature, humidity, and rainfall which may support rapid growth and development of mosquito vectors have been well reported in urban and peri-urban centers [8,11]. In Africa, malaria transmission is comparatively higher among the rural settings than the urban areas, which may be due to the higher vector density, poor housing status, and poor drainage systems in rural settings [8,12].

Hematological changes are other factors that may contribute to the transmission of microscopic malaria infection especially in children [7,13]. These changes may be related to the individual's packed cell volume, ABO blood grouping, and hemoglobin electrophoresis pattern [7,13]. Anemia has been known to be the usual sign of the parasitic infection in endemic malaria areas with *P. falciparum* being an important contributor to anemia in children [7,14]. Anemia is defined as a decrease in number of red blood cells (RBCs) or less than the normal quantity of hemoglobin for an individual's age and gender. From the age of puberty, hemoglobin of less than 10.0g/dl indicates anemia [14]. Resistance to malaria is characterized by the development of an immune response acquired by the host and also depends on innate features as protective factor against infections. Such innate features include ABO blood grouping, sickle cell trait (HbAS), and sickle cell disease (HbSS) [15]. The role of ABO blood group and the genetic make-up in adolescents susceptibility to malaria have not been fully studies [7,13-14]. Though, many studies on the association of malaria infection with ABO blood grouping and hemoglobin genotype have been reported in urban areas of Nigeria, majority of such studies were conducted in pregnant women and the under five children as the most vulnerable [7,8,13]. However, there is increasing evidence suggesting that school aged children and adolescents especially in rural settings are contributing to the number of population at risk of malaria [13,14]. Moreover, due to a decline in transmission and exposure in some areas, the peak age of clinical attacks of malaria is shifting from very young to older children and adolescents [13,15].

In order to adapt malaria control strategies to changes in transmission patterns in rural settings, there is urgent need for data on prevalence of *P. falciparum* infection and its associated factors among adolescent age groups in rural Nigeria. Identifying key risk factors are crucial for the effective implementation of preventive and health intervention programs. It is also essential for

policy formulation specifically for low-resource settings. Therefore, this study is aimed to determine the prevalence malaria infection among adolescent age groups and examine the associated determinants considering socio-demographic, Long Lasting Insecticide Nets Usage (LLINs) and hematological factors in rural community of Ekiti State, Southwestern Nigeria.

Materials and methods:

Study area/design/period: A hospital-based cross-sectional study was carried out between July 2021 and September 2022 at the federal teaching hospital, Ido-Ekiti (FETHI), Southwestern Nigeria. Ido-Ekiti is a rural community in Ekiti State and has a total land area of 332km2 and a total population of 159,114 inhabitants, according to the most recent population census conducted in 2006 [16]. The annual population growth rate is 3.2%, with the population in 2019 estimated to be 225,305 inhabitants [16]. Ido-Ekiti is located in a tropical rainforest with climatic and environmental conditions that support the growth of malaria transmission. The people are mainly farmers and traders in the informal sector with a relatively small portion of the working population and retirees in the formal sector [17]. In the study area, malaria transmission is perennial during the raining season (April – October) with *P. falciparum* being the major causative agent [17]. The health facility offers secondary and tertiary care to the people of its catchment area and its neighborhood states. The hospital has three outreach branches and was an accredited centre for postgraduate residency training in family medicine, pediatrics and other core clinical specialties. Recruitment procedure and collection of data were done by the trained resident doctors and medical officers at the family medicine, Igogo and Ado clinics, and adult emergency unit of FETHI..

Study population: This comprised all adolescent populations who presented to the family medicine, Igogo and Ado clinics, and adult emergency unit of the hospital.

Inclusion Criteria: Adolescent patients aged 10-19 years bracket who presented with febrile illness (with an axillary temperature $\geq 37.5^0$C) and included those who consented (aged 18 years and above) and those who assented by adults (below 18 years of age).

Exclusion Criteria: Patients who were too ill that required immediate attention or those with mental illness. Also, those on treatment for malaria or have just completed anti malaria within two weeks prior to the conduct of this study.

Sample size determination: This was determined using Araoye formular [18].

$n = \frac{Z^2 P(1-P)}{d^2}$ and $nf = \frac{n}{1+\frac{n}{N}}$ with a prevalence (P) of 84.2% [19] in a previous study on

ABO/Rhesus group and malaria parasite among children at 5% margin of errors

and 95% confidence interval. where n= minimum sample size when the population (N) of the participants in the study area was greater than 10,000 over a period of one year and nf is the minimum sample size when the population (N) of the participants was less than 10,000. From the year 2019 medical records of adolescent patients with malaria, N was 780 in 2020, Z= 1.96%.

$n = Z^2 P(1-P)/d^2 = (1.96)^2 \times 0.842 (1.000-0.842)/0.0025 = 204.$

Since $nf = \frac{n}{1+\frac{n}{N}} = 204/1+ (204/780)$

$= 204/1.416806 = 161.$

An attrition of 10% was added to cater for drop-out or loss of data and the minimum sample size was increased to 180 which was used for this study.

Sampling technique: Systematic random sampling technique was used in this study. The sampling frame is the total number of febrile adolescent individuals expected during the study period. Data from the records department for 2019 gave a sampling frame of 540 over a period of 14 (54 weeks) months. Dividing the sampling frame by the sample size (180) gave a sampling interval of four (3). On each screening day, the third registered respondent was selected by systematic random sampling technique. After that, every third respondent was selected. This process was repeated on each clinic day throughout the study period until the sample size of 180 was attained. Recruited individuals had their record cards tagged to prevent re-enrolment. The recruitment of the subjects was done by three trained resident doctors who served as research assistants while the researcher did the collection of data.

Ethical clearance, consideration and consent:

The study was approved by the Ethics and Research Committee of federal teaching hospital, Ido-Ekiti (ERC/2021/06/23/601A). When seeking consent from the respondents who were 18 year and above or assent from the guidance/parents of the respondent below aged 18 years, the methods and objectives of the study were explained clearly to the respondents individually. For those respondents that could not read or write, the questionnaire was translated from English language to their local language by an independent interpreter who served as their legal guardian while back translation to English language was done to maintain response consistency. The respondents were told that they were free to refuse or disengage participation at any time without losing any benefit of care or favor to those that participated. Thus, written informed consent either by appending signature or thumbprint was obtained from all adult respondents and guardians/parents on behalf of their children before starting the study. Confidentiality and privacy were ensured throughout the study. The study was at no cost to the respondents. All

malaria parasite individuals were treated with the standard medication according to the national malaria drug policy. The reporting of this study conforms to the strengthening the Reporting of observational studies in Epidemiology (STROBE) statement [20].

Data Collection Instruments: The two instruments for data collection were the interviewer administered questionnaire and data collection form. The questionnaire sought information about the respondents' socio-demographic characteristics (such as age, gender, education, occupation, and location), ownership of and sleeping under Long Lasting Insecticide Nets (LLINs).

Recruitment procedure: The data were collected using a pretested semi- structured interviewer administered questionnaire which was developed by the researcher. To ensure construct validity, the pretesting of the questionnaires was carried out on 10 febrile adolescent patients who were selected randomly at the family medicine clinic of nearby tertiary health facility. The pretesting was to see to the applicability of the instrument and recruitment procedure. Feedback from the pretest and validity assessment led to some modifications in the questionnaires. The questionnaires were translated from English to Yoruba language for those who did not understand English with the assistance of the interpreter while back translated to English language was subsequently done to maintain response consistency. The questionnaires took between 8 to 10 minutes to complete.

Clinical parameters of the respondents:

Sample collection: Samples of blood (about 5ml) were obtained intravenously with the assistance of hospital phlebotomist. The blood samples were transferred into an ethylenediaminetetraacetic acid (EDTA) bottle to prevent blood coagulation.

1. **Microscopy for malaria parasite**: Two blood films, one thin and one thick were made from the blood samples. The thick and thin smears were prepared on clean, dry

microscope glass slides and were allowed to dry. The thin smear was fixed in methanol and both smears were stained with 2% Giemsa BDH Laboratory supplies; Poole BH 15 ITD England [21]. The slides were viewed under a microscope using oil immersion at 100x magnification. Staining of the slides and parasite counting were done by a laboratory scientist of the hospital. The films were viewed for the presence of parasite. A positive result would mean presence of one parasite per 100 power field while a negative result would mean absence of parasite at 200 high power fields.

2. **Determination of genotype**: The genotype of each respondent was determined as described by Ochei and Kolhatkar [22]. About 1ml of blood samples were withdrawn from the EDTA bottle and centrifuged at 2500 rpm for 5 minutes. The supernatant was then discarded and packed cells were washed with normal saline for three times. The red cells were lysed by adding equal volume of distilled water, one quarter (1/4) of toluene followed by a drop of 3% KCN after the final wash. It was then mixed properly

3. **Determination of ABO blood group**. A drop of blood taking from the blood samples was placed on a clean slide in four concentric zones. Then, a drop of each of the anti-sera, anti-A, anti-B, and anti-D was added and mixed with each of the blood samples with the aid of a sterile stick. Blood groups were determined on the basis of agglutination as described by Rosenfield [23].

4. **Determination of Packed cell volume (PCV)**: A micro-haematocrit tube was filled with blood and centrifuge in a micro-haematocrit rotor at 10,000 rpm for 5 minutes. PCV was read using the micro-haematocrit reader, and recorded as, no anemia (PCV≥30%), mild anemia (25-29%), moderate anemia (20-24%), and severe anemia (<20%) [24].

Quality control: To ensure that the authorized standard operating procedure is followed for all the investigations, a senior scientist was recruited to examine the slide for quality control. Our methodology may be reproduce by fellow researchers if they so desire.

Treatment of the respondents: The results of blood film examination were given to the respondents. Respondents diagnosed with malaria and anemia were treated.

Data entry and analysis: Data collected were checked, cleaned and entered into EPI Info Version 7.0 and were exported to IBM SPSS for window version 21.0 (IBM Corp., Armonk, NY,USA), respectively for analysis. Quantitative data were expressed as mean ± standard deviation. Frequencies were used to determine the prevalence of malaria infection in the respondents. Binary logistic regression was employed to assess the determinants of *Plasmodium* infection. Variables significant at P-value < 0.05 in the univariate logistic regression were selected for multivariate logistic regression analysis model. Odds ratios with 95% confidence intervals were calculated and P-value< 0.05 was considered to be statistically significant.

Results:

A total of 180 respondents were screened during the study period. The distribution according to socio-demographic characteristics showed that the majority of the respondents 97 /180 (53.9%) were young children within the ages of 10-14 years. The mean age of the respondents was 14.5±2.7 years (range: 10-19 years). There were more males 94/180 (52.7%) than females 86/180 (47.8%). Many of the respondents were unemployed 125 (69.4%) and majority were secondary school holders 101 (56.1%), and were rural dwellers 139 (77.2%), (Table 1).

Table 1: Socio-demographic characteristics of respondents

Variable	Frequency (N = 180)	Percentage (%)
**Age (in years)**		
10 – 14	97	53.9
15 – 19	83	46.1
Mean age ± SD	*14.5 ± 2.7*	
Range	*10 – 19*	
**Sex**		
Male	94	52.2
Female	86	47.8
**Occupation**		
Farmer	16	8.9
Trader	18	10.0
Student	125	69.4
Artisan	21	11.7
**Education**		
None	8	4.4
Primary	47	26.1
Secondary	101	56.1
Tertiary	24	13.4
**Domicile**		
Rural	139	77.2
Urban	41	22.8

The majority of the respondents tested positive to *Plasmodium falciparum* 128/180; 71.1% (95% CI: 68.2%-73.8%), (Table 2).

Table 2: Prevalence of Malaria parasitaemia among respondents

**Variable**	**Frequency (N = 180)**	**Percentage (%)**
**Prevalence of Malaria parasitaemia**		
Positive	128	71.1
Negative	52	28.9
95% Confidence Interval	68.2%-73.8%)	

After adjusting for possible confounders; the odds of being infected with *P. falciparum* infection was 2.094 times (95% CI :1.288-3.403) higher among the respondents who had no formal education, 4.821 times (95% CI: 2.805 – 8.287) higher among the respondents who were rural dwellers, 1.950 times (95% CI: 1.525 –2.505) higher among the respondents who were not sleeping under LLINs, 3.420 times (95% CI: 1.003 – 11.657) higher among the respondents who were hemoglobin genotype A, 3.574 times (95% CI: 1.040 – 12.277) higher among the respondents who were genotype AS, 1.815 times (95% CI: 1.121 – 2.939) higher among the respondents who were rhesus positive, and 1.533 times (95% CI: 1.273 – 1.846) higher among the respondents who had severe anemia, (Table 3).

Table 3: Crude and Adjusted Odd Ratios for the factors significantly associated with malaria parasitaemia (N=180)

Variable	+ve %	-ve %	COR (95% CI)	p-value	AOR (95% CI)	p-value
**Occupation**						
Farmers	12(75.0)	4(25.0)	6.000 (1.407 –25.590)	0..012	2.250 (0.923 – 4.387)	0.234
Traders	11(61.1)	7(38.9)	3.143 (0.846 – 11.671)	0..083	1.833 (0.903 – 3.723)	0.158
Students	98(78.4)	27(21.6)	7.259 (2.664 – 19.779)	**<0.001**	2.352 (0.863 – 4.337)	0.234
Artisan (ref)	7(33.3)	14(66.7)	1.000		1.000	
**Education**						
None	6(75.0)	2(12.0)	4.200 (0.698 – 25.265)	0.102	2.094 (1.288-3.403)	**0.0.13**
Primary	41(97.2)	6(12.8)	9.567 (2.940 – 31.135)	**<0.001**	1.800 (0.969-3.345)	0.108
Secondary	71(70.3)	30(12.8)	3.133 (1.325 – 8.288)	**0.008**	1.687 (0.876 – 2.754)	0.127
Tertiary (ref)	10(41.7)	14(58.3)		1.000	1.000	
**Domicile**						
Rural	117(84.2)	22(15.8)	25 (11.363 – 55.621)	**<0.001**	4.821 (2.805 – 8.287)	**0.015**
Urban (ref)	11(26.8)	52(28.9)	1.000		1.000	
**LLITNs**						
Yes (ref)	36(46.2)	42(53.8)	1.000		1.000	
No	92(90.2)	10(9.8)	10.733 (4.871 – 23.650)	**<0.001**	1.950 (1.525 –2.505)	**0.003**
**Genotype**						
AA	95(76.0)	30(24.0)	11.083 (2.184 – 56.242)	**0.001**	3.420 (1.003 – 11.657)	**0.002**
AS	27(79.4)	7(20.6)	13.500 (2.282 – 79.880)	**0.001**	3.574 (1.040 – 12.277)	**0.004**
AC	2(25.0)	2(50.0)	1.167 (0.124 – 10.991)	0.893	1.125 (0.203 – 6.239)	1.000
SC	2(50.0)	2(50.0)	3.500 (0.284 – 43.163)	0.317	2.250 (0.470 –10.779)	0.726
SS (ref)	2(22.0)	7(77.8)	1.000		1.000	
**Blood group**						
O	63(76.8)	19(23.2)	9.118 (2.62 – 31.958)	**<0.001**	2.881 (0.734 – 6.725)	0.098
A	33(78.6)	9(21.4)	4.776 (1.333 – 17.111)	**0.012**	2.380 (0.832 – 5.648)	0.177
B	28(68.3)	13(31.7)	5.923 (1.582 – 22.172)	**0.005**	2.561 (0.927 –6.081)	0.141
AB (ref)	4(26.7)	11(73.3)	1.000		1.000	
**Rhesus factor**						
Rh D Positive	118(75.6)	38(24.4)	4.347 (1.785 – 10.587)	**<0.001**	1.815 (1.121 – 2.939)	**0.001**
Rh D Negative (ref)	10(41.7)	14(58.3)	1.000		1.000	
**PCV**						
≥ 30% (ref)	63(60.6)	41(39.4)	1.000		1.000	
25 – 29%	22(81.5)	5(18.5)	2.864 (1.004 – 8.164)	**0.043**	1.345 (0.657 – 1.706)	0.453
20 – 24%	17(81.0)	4(19.0)	2.766 (0.869 –8.806)	0.076	1.336 (0.453 –2.748)	0.127
< 20%	26(92.9)	2(7.1)	8.460 (1.905 – 37.579)	**0.001**	1.533 (1.273 – 1.846)	**0.001**

ref – reference category COR – Crude Odd Ratio AOR – Adjusted Odd Ratio

Discussion:

The study identified the prevalence of malaria infection and examined the socio-demographics, LLINs Usage and hematological parameters-based factors that determine the likelihood of malaria infection among adolescents aged 10-19 years in rural Southwestern Nigeria. The prevalence of 71.1% (95% CI: 68.2%-73.8%) found in this study revealed that the malaria infection was higher among the adolescent age groups. This finding was comparable to a cross sectional study conducted among school aged participants in Akure Southwestern Nigeria (71.7%) [19], and Abia Southeastern Nigeria (68.1%)[25]. This similarity in the prevalence might be due to the use of bed nets and gradual development of immunity, to a lesser extent, outdoor transmission [8,11]. In contrast, this study showed a higher prevalence of malaria than a cross sectional study conducted in Lagos Southwestern Nigeria (14.7%) [26], Kano Northwestern Nigeria (12.9%)[27], Kaduna Northcentral Nigeria (35.7%) [28] and Plateaus Northcentral Nigeria (36.6%) [25]. This could be due to the fact that while this current study was conducted during the peak of the raining season, the study in Lagos was conducted during the dry season, and other studies were conducted in Northern Nigeria. A study carried out across the six geopolitical regions of the country in 2010 concluded that the prevalence of malaria infection was higher in the Southwestern Nigeria compared to the Northern Nigeria [25]. Thus, differences in location, season variation, climatic conditions, and environmental factors might be responsible for this finding [9]. Furthermore, the prevalence of malaria in this study was lower than a cross sectional study conducted among first year student in Southeastern Nigeria (80.0%) [29], and in Southern Tanzania (78.0%) [30]. The low prevalence in this study compared to the other previous malaria study in Awka, Southeastern Nigeria might be attributed to the usage of insecticide treated nets as well as the setting. However, it is noteworthy to state that malaria

prevalence recorded in this study is of public health importance and could be attributed to several factors in the study area such as climatic, vegetation and environmental factors and the increase chances of participants contact with malaria.

Furthermore, our finding revealed that *P. falciparum* was the only plasmodium species encountered in this study. This is comparable to cross sectional studies conducted in Ekiti [31], and Akure [19], Southwestern Nigeria, and other malaria endemic setting [14]. This finding was not surprised given the fact that *P. falciparum* is the dominant *Plasmodium* species in Southwestern Nigeria [19,31], and in Africa [32], and 99.7% estimated malaria cases of *P. falciparum* have been documented from WHO African regions [1]. It is responsible for the reported high morbidity and mortality among children, especially in sub-Sahara Africa countries. Using multivariate regression for factors that were significantly associated with malaria infection in this study, respondents who had no formal education were 3.403 times more likely to increase the odds of malaria infection as compared to those who had formal education. This was similar to cross sectional studies conducted in Ibadan Southwestern Nigeria [11], Kano Northwestern Nigeria [9], and Kenya [33] which showed that level of education significantly influences the knowledge, attitude, and practice of people in various malaria interventions, treatment, and control [33].

Similarly, the respondents who resided in the rural setting were 4.821 times more likely to increase the odds of malaria infection. This was in agreement with studies conducted in Ibadan Southwestern Nigeria [11] and Kenya [8]. However, other cross sectional studies conducted in Kano Northwestern Nigeria [27], and other Africa country [34] have reported a high prevalence of malaria infection among the urban residents. The increased odds of malaria infection among the rural dwellers in this study may be due to their increased exposure to constant mosquito bites

due to their poor housing and environmental factors. Other studies have attributed the increased odds of malaria infection in rural areas to lack of social mobilization concerning malaria prevention [8,33].

In this study, although, respondents reported ownership of a LLIN (71.7%), they do not consistently use it, as only 43.3% of the respondents reported to have been consistently sleeping under LLINs. This was in agreement with cross sectional studies conducted in various parts of Africa, where LLIN has been shown to be at its lowest among school children [7,11,25]. Prevention communication strategies geared towards transmission of ownership into usage are recommended. The use of LLINs in this study was found to be significantly associated with reduction of malaria infection as the results showed that respondents who failed to sleep under LLINs were 1.950 times more likely to have malaria infection as compared to the respondents who did. This finding was consistent with the reports of studies conducted in central Nigeria [7], Southwestern Nigeria [11], and western Kenya [35]. Appropriate utilization of LLINs is one of the major cost effective interventions adopted for the prevention of malaria transmission. This finding further reinforces the usefulness of LLINs and calls for its continuous awareness campaign to improve its usage in rural settings.

The study showed that respondents who were of hemoglobin genotype AA and AS were associated with increased odds of malaria infections. This was consistent with the reports of cross sectional studies conducted in Sokoto North Western Nigeria [14] and Osun, Southwestern Nigeria [36] which found hemoglobin AA and AS to be a predictors of malaria infection. However, reports by several other studies on the association between hemoglobin genotype and malaria infection have been inconsistent [13-15]. Study by Suchdev *et al* in Kenya had found no significant association between malaria infection and hemoglobin genotype AA,

AS, or SS [37]. While study by Jemimah *et al* found HbSS to have increased the risk of malaria infection [7], study by Amala *et al* in River State Nigeria found HbAS as most protective against severe malaria [15]. These variations in the reports by these several studies suggest that other factors like geographical, environmental, and climatic may be responsible for the transmission of malaria infection irrespective of the hemoglobin genotype [13,15,33].

This study also showed that rhesus positive respondents is the most dominant with 86.7% and was found to be 1.815 times more likely to increase the odds of malaria infection as compared to rhesus negative respondents. This finding was comparable with the reports of cross sectional study conducted in Okada Nigeria [38]. Although, there was no obvious reason for this outcome, the proportion of respondents with rhesus negative in this study (13.3%) should not be discarded, given its implications in abortion and the hemolytic disease of the new born [37].

In this study, anemia was found to be significantly associated with malaria infection. This finding was consistent with the reports of previous studies conducted among school aged populations in Abia State [25], and Osun State Nigeria [36]. However, study by Umaru and colleague in Kaduna State Nigeria found no significant association between anemia and malaria infection [28]. The mechanism through which malaria infection causes anemia is multi-factorials. In rural settings like the study area, as micronutrients deficiency is common in many malaria endemic settings, it is possible that these respondents may have been anemic initially, and malaria infection further added to the burden of anemia [28]. Our study showed that the increased prevalence of malaria infection among these adolescents, demonstrate that, a reduction in packed cell volume is associated with risk of malaria infection [28,36]. Physiologically, school aged children are susceptible to malaria infection due to their loss of acquired immunity, and thus increased likelihood of being anemic [28,32].

Limitations:

This study was based on cross sectional design and thus, had limited opportunities to measure any causal association between malaria infection and other factors. The sample size was small, therefore, findings may not represent the general population. The age and quality of the LLINs were not considered and information on its use was self-reported. Bed nets may have been torn or of poor conditions and therefore, failed to provide adequate protection, as has been previously reported in Kenya.[35] Hemoglobin level was only assessed at baseline, measurement of hemoglobin level for each suspected case could have provided a better assessment of the association between anemia and malaria in the study area.[35] Despite these limitations, the study generates distinctive information regarding the burden of, and associated risk factors for malaria infection among the adolescents, which could be helpful for formulating further steps to implement interventions in rural Southwestern Nigeria.

Conclusion:

The study showed a higher prevalence of malaria infection among the adolescent populations in rural Southwestern Nigeria. Lack of formal education, being a rural residence, not sleeping under LLINs, blood hemoglobin genotype AA and AS, rhesus positive individuals, and having severe anaemia were significantly associated with the risk of malaria infection. The study demonstrated the need to pay greater attention to adolescent populations in rural settings for malaria interventions and control programs. The results may assist the stakeholders in decision making to formulate further steps towards improved malaria prevention and control.

Acknowledgements: The authors would like to appreciate the participants, and also nurses, resident doctors and medical officers at the family medicine, outreach centers, and adult

emergency medicine departments and the management of FETHI where the study was conducted.

References:

1. World Health Organization. World Malaria Report 2018: Global malaria programme, WHO, Geneva.2018. https://www.who.int/malaria/publications/world malaria report 2018/en/.(Accessed 16 June 2021).

2. World Health Organization (WHO), World Malaria Report 2015. 20 Avenue Appa, 1211 Geneva 27, Switzerland: 2015.

3. Fana S, Bunza M, Anka S. et al. Prevalence and risk factors associated with malaria infection among pregnant women in a semi- urban community of north western Nigeria. Infect Dis Poverty.2015; AA-8. DOI:10.1186/s40249-015-0054-0

4. WHO. Malaria situation. Weekly epidemiological record 2015; 45 (90): 609-616.

5. Gething PW, Patil AP, Smith DL, Guerra CA, Elyazar IR, Johnston GL, et al. A new world malaria map: Plasmodium falciparum endemicity in 2010. Malar J .2011; 10: 378.

6. Fairhurst RM, Nayyar GM, Breman JG. Artemisinin-resistant malaria: Research challenges, opportunities, and public health implications. American journal of Tropical Medicine and Hygiene 2012; 87: 231-241.

7. Jemimah Y, Victor O, Elizabeth A, Akpu P, Lynda A. Plasmodium falciparum Infection among Febrile Patients Attending a Tertiary Health Facility in Central Nigeria: Prevalence, Hematologic and Sociodemographic Factors. Int J Trop Dis. 2019; 2(2): 1-6

8. Sultana M, Sheikh N, Mahumud RA, Jahir T, Islam Z. Prevalence and associated determinants of malaria parasites among Kenyan children. Trop Med Health 2017; 45: 25

9. Dawaki S, Al-Mekhlafi HM, Ibrahim IJ, Atroosh WM, Abdulsalam AM, Sady H et al. Is Nigeria winning the battle against Malaria? Prevalence, risk factors and KAP assessment among Hausa communities in Kano State. Malar J 2016; 15:351

10. Nmadu PM, Peter E, Alexander P, Koggie AZ, Maikenti JI. The prevalence of malaria in children between the ages of 2-15 visiting Gwarinpa General Hospital, life camp, Abuja, Nigeria. J Health Sci 2015; 5: 47-51.

11. Awosolu OB, Yahaya ZS, Haziqah MTF, Simeon-Oke IA, Fakunle C. A cross- sectional study of the prevalence, density, and risk factors associated with malaria transmission in urban communities of Ibadan, Southwestwern Nigeria. Heliyon 2021; 7: e05975.

12. Oladeinde BH, Omoregie R, Olley M. Malaria and anaemia among children in a low resource setting in Nigeria . Iran J Parasitol 2012; 7:31-7.

13. The Federal Republic of Nigeria. National Malaria Strategic Plan 2014-2020. Roll Back Malaria. 2014; 1-110. Available at www.nmcp.gov.ng. (accessed March 5 2021).

14. Osaro E, Jamiu MH, Ahmed HM, Ezimah ACU. Effect of plasmodium parasitaemia on some haematological parameters in children living in Sokoto, North Western, Nigeria. Int J Clin Med Res 2014;1(2): 57-64

15. Amala, Smart E, Nwibani, Chdiebere P. Malaria among pregnant women and children and the protective roles of ABO blood group and HB-genotype. Int J Dev Res 2015; 5:5317-5320

16. Slide share, author. Profile of Ekiti State.(18/3/2021). Available at www.slideshare.net/EkitiState

17. Amase N, Dayo EL, Hellen EA, Aliu AA. Malaria parasitaemia among pregnant women possessing freely donated insecticide treated nets (ITNs) in Ado Ekiti, Nigeria. Global Journal of Biology, Agriculture and Health Sciences. 2014; 3 (1): 86-90

18. Araoye MO, Subject Selection: Sample size determination In: Araoye MO. Research Methodology with Statistics for Health and Social Sciences. Nathadex Publishers: 2003; 117-118.

19. Awosolu O, Adesina F, Afolabi O, Ogunsanya D. Malaria parasite distribution and knowledge among students of Federal University of Technology, Akure, Nigeria. Animal Research International .2020; 17(3): 3903-3910

20. von Elm E, Altman DG, Egger M, Pocock SJ, Gotzsche PC, Vandenbroucke JP, et al. The strengthening the Reporting of observational studies in Epidemiology (STROBE) statement; guidelines for reporting observational studies. Ann Intern Med. 2007; 147: 573-7.

21. Monif GRG. Infectious disease: In: Obstetrics and gynaecology. Baker DA (ed.) 6th ed. Partenon, New York 2004: 280-286.

22. Ochei J, Kolhalkar A. Miscellaneous investigation in Haematology, Medical Laboratory Science, theory and practical data. McGraw-Hill Publishing Company Limited, India. 2008:314-330.

23. Rosenfield RG. Blood typing and associated immune-hematological test procedures.In: Noel R Rose. Manual of Clinical immunology. American Society for Microbiology, Washington, DC. 1976: 526.

24. Cheesbrough M. District Laboratory Practice in Tropical Countries. Low price edition, Publisher Cambridge University Press. 2010:240-241.

25. Noland GS, Graves PM, Sallau A, Eigege A, Emukah E, Patterson AE, et al. Malaria prevalence, anaemia and baseline intervention coverage prior to mass net distribution in Abia and Plateau States, Nigeria. BMC Infect Dis. 2014; 14: 168.DOI:10.1186/1471-2334-14-168.

26. Aina OO, Agomo CO, Olukosi VA, Okoh HI, Iwalokun BA, Egbunak N, et al. Malariometric survey of Ibeshe community in Ikorodu, Lagos State dry season. Malar Res Treat. 2013: 487250. Doi: 10.1155/2013/487250

27. Oladele OV, Onuoha SC, Hamafyelto HS, OMISOPE O, Fauziyya A, Akindigh M, et al. Prevalence of malaria infection among patients attending Murtala Muhammed Specialist Hospital, Kano, Nigeria. Afr J Clin Exper Microbiol. 2018; 19(3): 214-220. Doi: 10.4314/ajcem.v19i3.9

28.Umaru ML, Uyaiabasi GN. Prevalence of malaria in patients attending the general hospital, Makarfi, Kaduna State, North-western Nigeria. American J Infect Dis Microbiol. 2015; 3(1): 1-5.

29. Ibekwe AC, Okonko IO, Onunkwo AI, Ogun AA, Udeze AO. Comparative prevalence level of Plasmodium in Freshmen (first year students) of Nnamdi Azikwe University in Awka, South-Eastern, Nigeria. Malaysian Journal of Microbiology. 2009; 5(1): 51-54.

30. Harchut K, Standley C, Dobson A, Klaassen B, Rambaud-Althaus C, Althaus F, Nowak K. Over-diagnosis of malaria by microscopy in the Kilombero Valley, Southern Tanzania: an evaluation of the utility and cost-effectiveness of rapid diagnostic tests. Malar J. 2013; 12: 159. Doi:10.1186/1475-2875-12-159.

31. ibro

32. Hay SI, Guerra CA, Tatern AJ, Atkinson PM, Snow RW. Urbanization, malaria transmission and disease burden in Africa. Nat Rev Microbiol 2005; 3: 81-90

33. Imbahale SS, Fillinger U, Githeko A,et al. An exploratory survey of malaria prevalence and people's knowledge, attitude and practice of mosquito larva source management for mal;aria control in western Kenya. Acta Trop. 2010; 115: 248-56.

34. Kuesap J, Na-Bangchang K. The effect of ABO blood groups, hemoglobinopathy and heme oxygenase-1 polymorphisms on malaria susceptibility and severity. Korean J Parasitol. 2018; 56: 167-173.

35. Kepha S, Nikolay B, Nuwaha F, Mwandawiro CS, Nankabirwa J, Ndibazza J et al. Plasmodium falciparum parasitaemia and clinical malaria among school children living in a high transmission setting in western Kenya. Malar J. 2016; 15: 157.DOI: 10.1186/s/12936-016-1176-y

36. Egbewale BE, Akindele AA, Adedokun SA, Oyekale OA. Prevalence of asymptomatic malaria parasitaemia and anaemia among elderly population in Osun State, Southwestern Nigeria. Inter J Comm Health Publ Health. 2018; 5(7): 2650-56.

37. Suchdev PS, Ruth LJ, Earley M, Macharia A, Williams TN. The burden and consequences of inherited blood disorders among young children in western Kenya. Matern Child Nutr. 2014; 10(1): 135-144.

38. Otajevwo FD. Prevalence of malaria parasitaemia and its association with ABO blood grouping among students of Igbinedon University, Okada,Nigeria. BJMMR 2013; 3:1164-77

Data

Click here to access/download
Supporting Information
Malaria Data 180 cases.xlsx
