# Supplementary material for: Malaria infection and its association with socio-demographics, long lasting insecticide nets usage and hematological parameters among adolescent patients in rural Southwestern Nigeria
Source: PLoS One. 2023 Jul 14;18(7):e0287723. doi: 10.1371/journal.pone.0287723 (PMC10348556; doi:10.1371/journal.pone.0287723)
Supplement: Attachment — Submitted filename: Summary of reviewer.docx [file pone.0287723.s004.docx]

**Summary of reviewer’s comments and Authors’ responses. (PONE-D-23-02179)**

| **Reviewer’s comments** | **Authors’ Response** |
| --- | --- |
| **Editor’s comment**:  In addition to the comments raised by the reviewers, the authors will need to re-work the data analysis in Table 3, particularly the percentages and carefully go through the entire manuscript to correct grammatical errors as well as italicize all scientific names.  If you would like to make changes to your financial disclosure, please include your updated statement in your cover letter. Guidelines for resubmitting your figure files are available below the reviewer comments at the end of this letter.  **Journal requirements**:   1. Please ensure that your manuscript meets PLOS ONE’s style requirements. 2. In the ethics statement in the Methods, you have specified that verbal consent was obtained. Please provide additional details regarding how this consent was documented and witnessed, and state whether this was approved by the IRB   3.We noticed you have some minor occurrence of overlapping text with the following previous publication(s), which needs to be addressed:  In your revision ensure you cite all your sources (including your own works), and quote or rephrase any duplicated text outside the methods section. Further consideration is dependent on these concerns being addressed.  4. Thank you for stating the following financial disclosure:  “Self funded by authors”  At this time, please address the following queries:  a) Please clarify the sources of funding (financial or material support) for your study. List the grants or  organizations that supported your study, including funding received from your institution  .  b) State what role the funders took in the study. If the funders had no role in your study, please state: “The funders had no role in study design, data collection and analysis, decision to publish, or preparation of the manuscript.”  c) If any authors received a salary from any of your funders, please state which authors and which funders.  d) If you did not receive any funding for this study, please state: “The authors received no specific funding for this work.”  Please include your amended statements within your cover letter; we will change the online submission form on your behalf.  5. Thank you for stating the following in your Competing Interests section:  “No competing interest”  Please complete your Competing Interests on the online submission form to state any Competing Interests. If you have no competing interests, please state "The authors have declared that no competing interests exist.",  This information should be included in your cover letter; we will change the online submission form on your behalf.  6. We note that you have stated that you will provide repository information for your data at acceptance. Should your manuscript be accepted for publication, we will hold it until you provide the relevant accession numbers or DOIs necessary to access your data. If you wish to make changes to your Data Availability statement, please describe these changes in your cover letter and we will update your Data Availability statement to reflect the information you provide.  **5**. **Review Comments to the Author**  **Reviewer #1**  The discussion needs a major review, for instance there was no basis for comparing the prevalence of this study to those whose patient selection was not similar. The result of this study cannot be compared to that of Akure or Lagos where the study populations were apparently health and of different ages.  -Outdoor transmissions or development of immunity were not assessed in this study, so they cannot be implied as stated in the discussion. Same with exposure to constant mosquito bites in participants, which was assumed.  Authors should indicate if prevalence of malaria parasitaemia in table 2 was derived from thick film or thin film? Findings from thick film and thin film were not clearly stated in the result section.  Some few grammatical errors:- First line of quality control, “is” should be changed to “was”, Sides not slide, reproduced not reproduce  Regarding the treatment of respondents: Better to clarify that the results were “transmitted to the managing physicians through the patients” than to just write that test results were given to respondents  How was the P. falciparum specie determined in this study?  Limitations- Self report of too many limitations is not good, especially when some of them are avoidable. Sample size should not be a limitation since it was scientifically derived  Failure to find out the quality of LLITNs is an admission of negligence by the research team, rather than limitation. Better to keep quiet about this.  The conclusion of high malaria prevalence in this study area cannot be safely derived from febrile subjects. This gives a impression that the subjects were apparently healthy.  Overall, this is a good study that is worthy of attention if the results and discussion are well revised  **Reviewer #2:**  1. Its difficult for me to trust the microscopy results. Since this is the only method used to confirm Malaria parasite, it would have been nice to follow a standard microscopy procedure. Two microscopists should have done the reading with the third microscopists confirming any discrepancies arising from the reading of the two initial microscopists. Unfortunately, this was not done and that spurs me to recommend rejection.  2. For the calculation of the odd ratio, I feel also that the control group is very small for any meaningful inference.  3. The limitations listed in the manuscripts do not look like an insurmountable challenges but shows that the work was not well planned.  5. I strongly recommend the work to other lower journals where the public could still be reached with the outcome of the research.  6. I am worried that the author did not provide the parasite density results of the microscopy, at least to see to what degree the variables could influence parasite density.  7. The author mentioned that this is a cross-sectional study but none of the results were presented to indicate it so.  8. The -ve and +ve in Table 3 is very confusing. The legend could not provide what they stood for.  **Reviewer #3**:  **Minor comments**  Introduction  The WHO epidemiological record and others references should be updated (for example, instead of WHO 2015 report use the actual report!)  Materials and methods  The determination of the genotype was not completely described. This should be done.  Results  - Table 2: the parenthesis should be completed;  ggg  - Table 3:  The wrong percentages for Domicile Urban (Ref) and genotype AC should be corrected;  What are the selection criteria of Ref. for each variable? This should be well explained in the methodology;  The age and sex should also be considered since the authors have divided the population into two groups (see Table 1)  Limitations:  The lack of children less than 10 years old is also a possible limitation for having a reel burden of malaria in these older children and adolescent.  References:  In the section “References”: the ref. 6, 8, 9, 10, 11, 12, 14, 15, 23, 31, 32should be corrected.  END | Thank you for the efforts you put in to improve our manuscript. Your contributions are highly appreciated. Please find our responses below.  ---The data has been reviewed and errors have been corrected. Check table 3 page 13. All scientific names have been italicized.  -The authors did not receive any funding for this work. This has been included in the cover letter.  1.The style requirement has been strictly followed.  2.Verbal consent was not specified in the manuscript. Rather, written informed consent was taken. Please see ethical consent and consideration under methodology in our first submission. The same was repeated in the revised manuscript on page 7, line 150  3.Minor occurrence of overlapping with previous publications have been identified, and the statements have been modified. Appropriate references cited. See  Page 14, line 285-287:Page 15 line 308-309: Page 16, line 329-332: page 18, line 369-372, 379-380  4.The authors received no specific funding for this work.  This has been included in the cover letter.  5.The authors have declared that no competing interests exist. This has been included in the cover letter.  6.The data for this study will be made available as supporting information upon acceptance of this manuscript. This has been included in the cover letter.  Thank you for the efforts you put in to improve our manuscript. Your contributions are highly appreciated. Please find our responses below  The discussion has been reviewed. Comparisons of prevalence whose selections were not similar have been removed. See page 14, line 278-283,  This statement “Outdoor transmissions or development of immunity” and “exposure to constant mosquito bites in participants” and their citations have been removed’ Page15, line 295  These comments have been addressed in table 2. See table 2. Page 12, line 251-253  Grammatical errors under methodology were noted and corrected. Page 10, line 215-217  This statement has been modified. See page 10, line 218-220 .  The determination of *P.falciparum* specie has been clearly explained on page 9, line 184—197  Some of the limitations have been removed. Page 18 line 364-372  The conclusion has been modified. Page 18, line 374-380  1.The missing information has been corrected on page 9,line 184-197  2.This was part of the limitations.  3.Limitations have been modified on page 18 line 364-372  6. Parasite density has been included in Table 2. Page 12, line 254-255  8. The legend has been provided in Table 3. Line 254-255.  The affected sentences in the introduction have been updated including the references. See page 3, line 58-63 and references 2-6 under references.  The description of genotype determination has been modified. See page 9, line 204-205  Table 2 has been modified page 12.  The percentages for Urban and genotype AC have been corrected. Check table 3, page 13  For selection criteria of Ref, check page 10, line 228-229.  The age and sex have been included. Check table 3, on page 13  This statement has been added to the limitation. Check page 18, line 265-267.  All the quoted references have been corrected or replaced. See references.  END |
