# Supplementary material for: Malaria infection and its association with socio-demographics, long lasting insecticide nets usage and hematological parameters among adolescent patients in rural Southwestern Nigeria
Source: PLoS One. 2023 Jul 14;18(7):e0287723. doi: 10.1371/journal.pone.0287723 (PMC10348556; doi:10.1371/journal.pone.0287723)
Supplement: Attachment — Submitted filename: Review 2023 (2).docx [file pone.0287723.s006.docx]

**Review**

Malaria remains a public health problem. The impacts of different interventions against malaria such as a decline in transmission and exposure in some areas have been recorded, and the peak age of clinical attacks of malaria is shifting from very young to older children and adolescent.

In order to adapt malaria control strategies to changes in transmission patterns, there is urgent need for data on prevalence of *P. falciparum* infection and its associated factors among adolescent age groups which are crucial for the effective implementation of preventive and health intervention programs. Thus, the authors have raised an important issue on determining the prevalence malaria infection among adolescent age groups and examine the associated determinants considering socio-demographic, Long Lasting Insecticide Nets Usage (LLINs) and hematological factors in rural community of Ekiti State, Southwestern Nigeria.

**Minor comments**

**All small errors are highlighted in the document provided.**

**Results**

- Table 3:
- The wrong percentages for Domicile Urban (Ref) should be corrected;

**References**

In the section “References”:

- the ref. 3, 8, 17, 19, 24, 29, should be corrected.
- Line 453-455: font should be corrected.
