# Supplementary material for: Malaria infection and its association with socio-demographics, long lasting insecticide nets usage and hematological parameters among adolescent patients in rural Southwestern Nigeria
Source: PLoS One. 2023 Jul 14;18(7):e0287723. doi: 10.1371/journal.pone.0287723 (PMC10348556; doi:10.1371/journal.pone.0287723)
Supplement: Attachment — Submitted filename: SUMMARY OF REVIEWERS.docx [file pone.0287723.s008.docx]

**SUMMARY OF REVIEWERS’ COMMENTS AND AUTHOR RESPONSE (PONE-D-23-02179R1]**

**Author’s opening remark:** I appreciate the academic editor and other reviewers at improving the quality of our work. Please find my responses to your comments and suggestions below.

| **Academic editor’s comment** | **Author’s response** |
| --- | --- |
| --**In table 2 for example**, Malaria parasitaemia was classified as mild, moderate and severe parasitaemia in 128 individuals who tested positive for *P. falciparum*. However, the frequency N was erroneously taken as the overall participants enrolled (180) rather than the total number positive for *P.falciparum* (128). Consequently, the percentages will not add up to 100%.  **--In table 3**, percentages were generally calculated across rows rather than across columns. Since you are comparing between positives and negatives, the percentages (as well as statistical analyses should be calculated /compared across column rather than across rows. For example, a reader may wish to know the impact of using LLITNs on malaria by comparing the proportion of those using LLITNs among participants who tested positive, with the proportion of those using LLITNs among participants who tested negative.  **--In addition**, under domicile in table 3, the urban percentages (+ve and –ve) do not add up to 100%. Please, check and correct.  **Journal Requirements**:   1. Please review your reference list to ensure that it is complete and correct. If you have cited papers that have been retracted, please include the rationale for doing so in the manuscript text, or remove these references and replace them with relevant current references. Any changes to the reference list should be mentioned in the rebuttal letter that accompanies your revised manuscripts. If you need to cite a retracted article, indicate the articles retracted status in the references list and also include a citation and full reference for the retraction notice. 2. **Additional editor comments:**   **Introduction:**   - **P**age 3: lines 57 and 58. Please remove italics from “genus” - - Correct the clause” of which *P.falciparum* being the most prevalent “to –of *which P. falciparum* is the most prevalent” - Page 3, line 57 and 58. - Please review the statement “Hematological changes are other factors that may contribute to the transmission of microscopic malaria infection especially in children. Hematological profile may contribute to the clinical presentation of malaria, but not transmission of malaria infection.   **-**Page 4, line 96:  Please, correct the clause “determined the prevalence malaria infection ---“to determined the prevalence of malaria infection”  **Discussion:**  **Page 14; LINE 278**  Please correct “higher” to high” since there was no other group for comparison.  **Page 15, line 297**  **-**Plasmodium should be uppercase letter. Please correct “*plasmodium species* “to” *Plasmodium species*”  **Reviewer#3:**  All small errors are highlighted in the document provided.  **Results:**  **Table 3:** The wrong percentages for domicile Urban (ref) should be corrected.  **References:**  In the section “References”, -the ref. 3,8,17, 24, 29 should be corrected.  -Line 453-455: font should be corrected.  **Reviewer #4:**  **P**lease check my comments in the text especially the discussion section which should be revisited as it does not appropriately address the content of your study.  **END#** | Thank you for this observation.  The correction has been effected in table 2, page 12, line 246  -This has been corrected in table 3, page13. All percentages were calculated/compared across columns.  -The correction has been effected in table 3, page 13.  -The reference list has been reviewed. The new references that replaced the previous references were highlighted in pink color under our revised manuscript with track changes. See the reference list  This has been corrected on page 3, Line 53 under the introduction  The correction effected on page 3, line 53-54  The statement has been reviewed. See page 3, line 70-72  -This statement has been modified on page 5, line 95  --This has been corrected on page 14, line 272 , line  ----This has been corrected on page 15, line 289  All small errors were corrected and highlighted in the manuscript. See the following pages;  Page 3, line 53,Page 4, line 84, Page 8,line 167, page 10 line 205, page 11 line 232, page 16, line 311, page 16, line 324  This has been corrected, See table 3, page 13  Some of the references (including 3,8,17,24,29) have been corrected while some were replaced with new and current references. These were highlighted in pink color in the reference list in the manuscript.  This has been corrected.  Some paragraphs in the discussion have been re-written and some sentences were replaced. These affected paragraphs and sentences were highlighted in pink color in the manuscript.  These comments have been adequately addressed. Some areas of the discussion as mentioned in the text have been re-written. See page 14, line 269, line 272-275.  Page 16, line 324-344,  Page 17, line 349-355.  **END#** |
